# Curcumin and its analog alleviate diabetes-induced damages by regulating inflammation and oxidative stress in brain of diabetic rats

**DOI:** 10.1186/s13098-021-00638-3

**Published:** 2021-02-18

**Authors:** Chengfeng Miao, Hanbin Chen, Yulian Li, Ying Guo, Feifei Xu, Qi Chen, Yanyan Zhang, Mengjun Hu, Guorong Chen

**Affiliations:** 1 Department of Pathology, The First Affiliated Hospital of Wenzhou Medical University, Wenzhou, Zhejiang PR China; 2 Department of Radiation Oncology, The First Affiliated Hospital of Wenzhou Medical University, Wenzhou, Zhejiang PR China; 3 Department of Pathology, The Second Affiliated Hospital and Yuying Children’s Hospital of Wenzhou Medical University, Wenzhou, Zhejiang PR China; 4 Department of Pathology, Zhuji People’s Hospital, Shaoxing, Zhejiang PR China

**Keywords:** Diabetes mellitus, Brain, Curcumin analog, Inflammation, Oxidative stress

## Abstract

**Background:**

Diabetic encephalopathy is a severe diabetes complication with cognitive dysfunction and neuropsychiatric disability. The mechanisms underlying diabetic encephalopathy is believed to be relevant with oxidative stress, vascular amylin deposition, immune receptors, inflammation, etc. This study wanted to evaluate the ability of curcumin and its analog A13 to alleviate oxidative stress and inflammation in diabetes-induced damages in brain.

**Methods:**

Sixty adult male Sprague–Dawley rats were divided into 5 groups: normal control (NC) group, diabetes mellitus (DM) group, curcumin-treated diabetes mellitus (CUR) group, high dose of A13-treated diabetes mellitus (HA) group, low dose of A13-treated diabetes mellitus (LA) group. Activation of the nuclear factor kappa-B (NF-κB p65) pathway was detected by RT-qPCR, immunohistochemical (IHC) staining and Western blot; oxidative stress was detected by biochemical detection kit; brain tissue sections were stained with hematoxylin–eosin (HE) staining and Myelin staining.

**Results:**

RT-qPCR, IHC staining and Western blot showed that curcumin and A13 treatment could inhibit the NF-κB p65 pathway. Curcumin and A13 increased the activity of superoxide dismutase and decreased the malondialdehyde level in the brain of diabetic rats. Furthermore, HE staining and Myelin staining demonstrated that the histological lesions of the brain in diabetic rats could be significantly ameliorated by curcumin and A13.

**Conclusion:**

Curcumin analog A13 could alleviate the damages in the brain of diabetes rats by regulating the pathways of inflammation and oxidative stress. A13 may be a new potential therapeutic agent for diabetic encephalopathy.

## Background

Diabetes mellitus (DM) is a common chronic endocrine disease. It was estimated that there would be over 600 million people living with type 2 diabetes worldwide in 2045 [1]. Without adequate treatment and control, the patients would have multiple system complications (such as diabetic cardiopathy, diabetic nephropathy, diabetic encephalopathy, etc.) [2]. In the 1950s, the term ‘diabetic encephalopathy’ was first introduced to describe central nervous system related complications of diabetes [3]. Because of the diabetes-related microvascular damages and the changes of microenvironment, patients with type 2 diabetes have lots of neurological disabilities, including worse learning, processing speed, memory and attention compared to individuals without diabetes [4, 5]. Some studies also showed that diabetic encephalopathy was significantly associated with Parkinson’s disease, acute ischemic stroke (AIS) and other brain ischemic injuries [6, 7].

The mechanisms underlying diabetic encephalopathy had been believed to be relevant with vascular amylin deposition [8], oxidative and nitrative stress [9], inflammation, hyperglycemic internal environment [10, 11], endoplasmic reticulum (ER) stress, autophagy defects, and immune receptors [12]. Wang, Z found that the inflammatory response related proteins were significantly increased in the brain with diabetes, including tumor necrosis factor-α (TNF-α), NF-κB, cyclooxygenase-2 (Cox-2) and interleukin-6 (IL-6) [13]. In mammals, NF-κB family has five different members including p65 (RelA), RelB, c-Rel, p50/p105 (NF-κB1) and p52/p100 (NF-κB2). NF-κB p65 is the most common and the most evaluated member of NF-κB family [14]. The activated NF-κB p65 protein had been found in the cerebral cortex, hippocampus and hypothalamus [15].

**Fig. 1:**
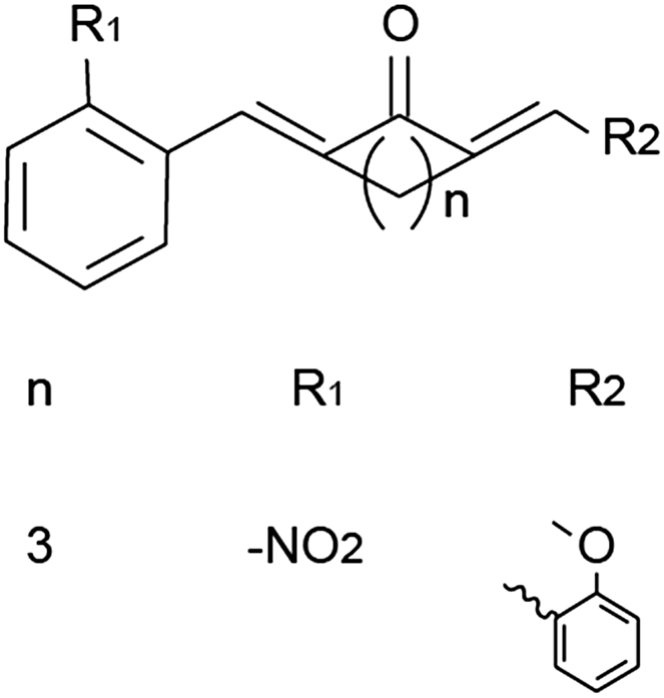
Molecular structure of curcumin analog A13

Curcumin, the traditional medicine in China and India, is a natural phytochemical ingredient from the root of curcuma [16]. Several lines of evidences indicated that curcumin could treat a wide variety of diseases including diabetes complications, chronic inflammation, hyperlipoidemia, neurodegenerative diseases, amenorrhea, etc. [17]. Meanwhile, curcumin even had low or no toxic side effects [18]. However, the curcumin’s bioavailability was very poor because of its insolubility in water, low absorption and rapid metabolism in digestive system [19, 20]. Different methods had been proposed to improve its bioavailability, like complexing with piperine or metal ions [21], using PLGA nanoparticles [22], using poly-ε-caprolactone nanoparticles [23], using curcumin nanoparticles [24], etc. Nonsteroidal mono-carbonyl curcumin analog A13 (Fig. 1) without the β-diketone moiety, had an enhanced stability in vitro and an improved pharmacokinetic profile in vivo [25, 26]. Previously, our lab demonstrated that curcumin analog A13 alleviates oxidative stress in the myocardium of high-fat-diet and streptozotocin-induced diabetic rats [27]. In this study, we used curcumin and its analog A13 to compare their therapeutic effects on the NF-κB p65 pathway in the brain of diabetes rat. In addition, we also researched the change of oxidative stress indicators in the brain of diabetes rat.

## Materials and methods

### Reagents and antibodies

Curcumin was purchased from Sigma-Aldrich Co (St. Louis, MO, USA). Curcumin analog A13 was the gifts from professor Guang Liang in Wenzhou Medical University (Wenzhou, China). The antibodies for Western bolt and immunohistochemistry include NF-κB p65 (Cell Signaling Technology, Cat#8242), β-Actin (Cell Signaling Technology, Cat#4970), Cox-2 (Cell Signaling Technology, Cat#12,282), TNF-α (Affinity Biosciences, Cat#AF7014), Histone H3 (Abcam, ab1791). We bought the Superoxide Dismutase (SOD) Detection Kit (A001; Nanjing Jiancheng Bioengineering Institute, Nanjing, China) and Malondialdehyde (MDA) Detection Kit (A003; Nanjing Jiancheng Bioengineering Institute, Nanjing, China) to examine the activity of SOD and the level of MDA.

### Experimental animals

Sixty SPF-rated, male, Sprague–Dawley rats were kept in standard laboratory conditions of temperature (20 ± 2 ℃) and humidity (55 ± 5%). 60 Rats were randomly divided into 2 groups (10 rats for control group, the others for experimental group). The experimental group was fed with a high-fat diet for four weeks while the NC group received a standard diet. After four weeks, the diabetic model in the experimental group was established by intraperitoneal injection of streptozotocin (30 mg/kg). Streptozotocin for establishing the rat model of diabetes was dissolved in citrate buffer (pH = 4.4). Meanwhile, NC group was injected with the carrier vehicle alone. After four weeks, the diabetes model with fasting blood glucose detection (FBG) ≥ 16.7 mmol/L was considered successfully established. Successful models were randomly divided into the following groups: DM group (n = 12), CUR group (n = 10), HA group (n = 10), LA group (n = 10). In the next 8 weeks, the intragastric administration was given once a day. Curcumin and A13 were dissolved in double distilled water containing 1%CMCNa. Building upon our lab experience of curcumin and A13 for the treatment of diabetic rats [27], the CUR group rats were given curcumin 20 mg/kg. The HA group rats were intragastrically administered A13 at a dose of 20 mg/kg, and the LA group rats were 10 mg/kg. Meanwhile, the NC group and DM group received an equal volume of of 1% CMCNa solution. Body weight and FBG levels were measured every week.

### Preparation of tissue samples

At the end of the experiment, we injected the 10% chloral hydrate into peritoneum of rat at a dose of 0.3 ml/100 g for anesthesia. Sacrificed by bloodletting, the brain tissue was quickly taken out. Part of cerebral cortex tissue was placed in nitrogen canister for quantitative test later. Partial brain and cerebellum tissues were fixed in 10% neutral formalin for HE staining, immunohistochemistry and Myelin staining. All operations were performed on ice.

### RT-qPCR

Total RNA of cerebral cortex was isolated using TRIzol (Ambion, USA). According to the manufacturer’s instruction, cDNA was synthesized by using the PrimeScript™ RT Reagent Kit (Takara, RR037A, Japan) in a total volume of 10 μl. The resultant cDNA was performed with The QuantStudio® 5 Real-Time PCR system (Thermo Fisher Scientific, USA) using TB Green™ Premix Ex Taq™ II (Takara, RR820A, Japan). The sequences of primers for RT-qPCR were listed in Table 1.

**Table 1:** Primers for myocardium genes

Gene	Forward and reverse primers
*Nf-κb p65*	F: 5’-TGG CTT CTA TGA GGC TGA ACT CTG-3’
	R: 5’-TTG CTC CAG GTC TCG CTT CTT CTT C-3’
*Tnf-α*	F: 5’-GGT ATG AAA TGG CAA ATC G-3’
	R: 5’-GCA AAC CAC CAA GCA GAG-3’
*Cox-2*	F: 5’-CTT CCT CCT GTG GCT GAT GAC TG-3’
	R: 5’-GGT CCT CGC TTC TGA TCT GTC TTG-3’
*Sod* *Gapdh*	F: 5’-CCA CGA GAA ACA AGA TGA CT-3’
	R: 5’-GAC TCA GAC CAC ATA GGG AAT-3’ F: 5’-CCT TCC GTG TTC CTA CCC-3’ R: 5’-AAG TCG CAG GAG ACA ACC-3’

The relative mRNA levels of *Nf-κb p65*, *Tnf-α*, *Cox-2* and *Sod* was calculated by the 2 ^–ΔΔCT^ method. Then, the mRNA level of target gene was normalized with reference to expression of *Gapdh*.

### Western blot analysis

Total protein was conducted from the frozen tissues of cerebral cortex using RIPA buffer (50 mM Tris (pH 7.4), 150 mM NaCl, 1% Triton X-100, 1% sodium deoxycholate, 0.1% SDS) containing 1% protease inhibitors. Nucleoprotein extraction was conducted using nuclear and cytoplasmic protein extraction kit (product code: P0028; Beyotime Biotechnology, Inc, Shanghai, China). Then, the protein concentration was determined by BCA Protein Assay Kit (Beyotime, P0010). And an equal amount of protein from each sample was separated by SDS-PAGE gels and transferred onto the PVDF membranes. After blocking with 5% milk in TBST for 2 h, the membranes were reacted overnight at 4℃ with antibodies, including anti-β-Actin antibody, anti-Histone H3 antibody, anti-NF-κB p65 antibody, anti-TNF-α antibody and anti-Cox-2 antibody. Subsequently, the membranes were washed extensively and incubated with Goat anti-rabbit IgG (Bioworld Technology, USA, 1:1000). Finally, the level of protein was determined and analysed by Image Lab (Bio-Rad Laboratories, USA). We used Histone H3 as an internal control for nucleoprotein loading and β-Actin as an internal control for total protein loading to normalize each sample.

### HE staining, IHC staining and Myelin staining

The specimens of brain and cerebellum were fixed in 10% buffered formaldehyde. Then, the sample was dehydrated, embedded and sectioned. Sections (3.5 μm) were used for immunohistochemical staining, HE staining and Myelin staining later. In immunohistochemical staining, the primary antibodies included anti-NF-κB p65 antibody and anti-TNF-α antibody. Furthermore, we made the Myelin staining with Luxol Fast Blue/Cresyl Violet Stain Kit (G3245; Solarbio, Beijing, China) according to the manufacturer’s instruction. All the other chemical reagents used in this study were of analytical grade.

### Statistical analysis

Data were presented as mean ± SD. For comparing the differences in 5 groups, data were evaluated by one-way ANOVA test using SPSS 22.0 (IBM, New York, USA) software. GraphPad Prism software package V6.0 was used to draw bar chart. Results were considered as statistically significant with a *P*-value < 0.05.

## Results

### Effects of curcumin analog A13 on weight and plasma glucose

**Fig. 2:**
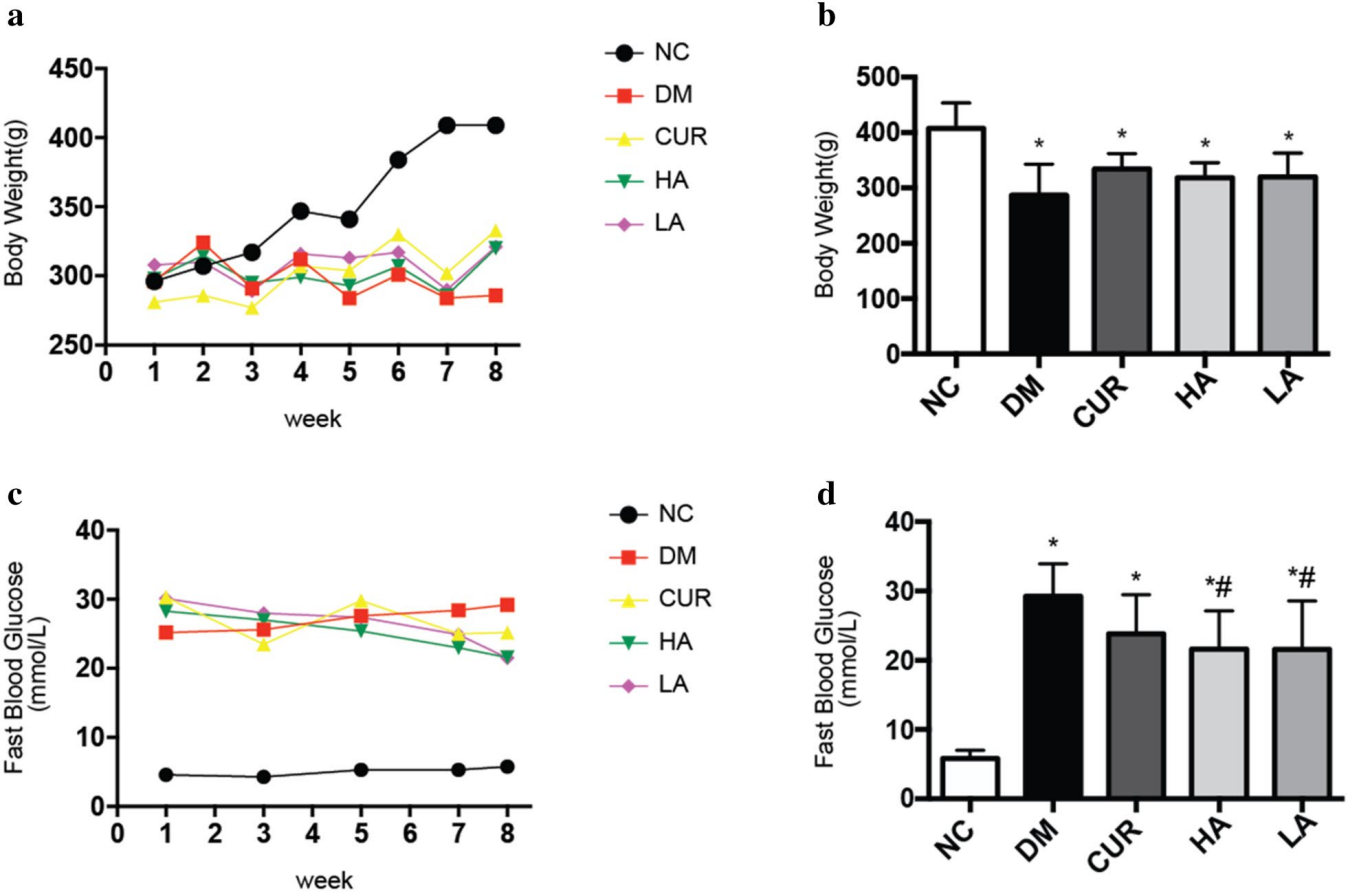
The body weight and blood glucose in 8 weeks after establishment of groups (**a**, **c**). The body weight and fast blood glucose at the end of the experiment (**b**, **d**). *P < 0.05 versus the NC group; #*P* < 0.05 versus the DM group

At the end of the experiment (Fig. 2), the blood glucose of DM group (29.25 ± 4.68 mmol/L) was increased obviously as compared to NC group (5.81 ± 1.18 mmol/L). And A13 could decrease the blood glucose of DM group significantly (*P* < 0.05). Meanwhile, the body weight of DM group was decreased compared with NC group (*P* < 0.05). Although, the weight of three treatment groups didn’t restore to the healthy level, curcumin or A13 could increase weight of DM group.

### Curcumin analog A13 delayed histological lesions and inhibit NF-κB p65 pathway in the brain of diabetes rats

**Fig. 3:**
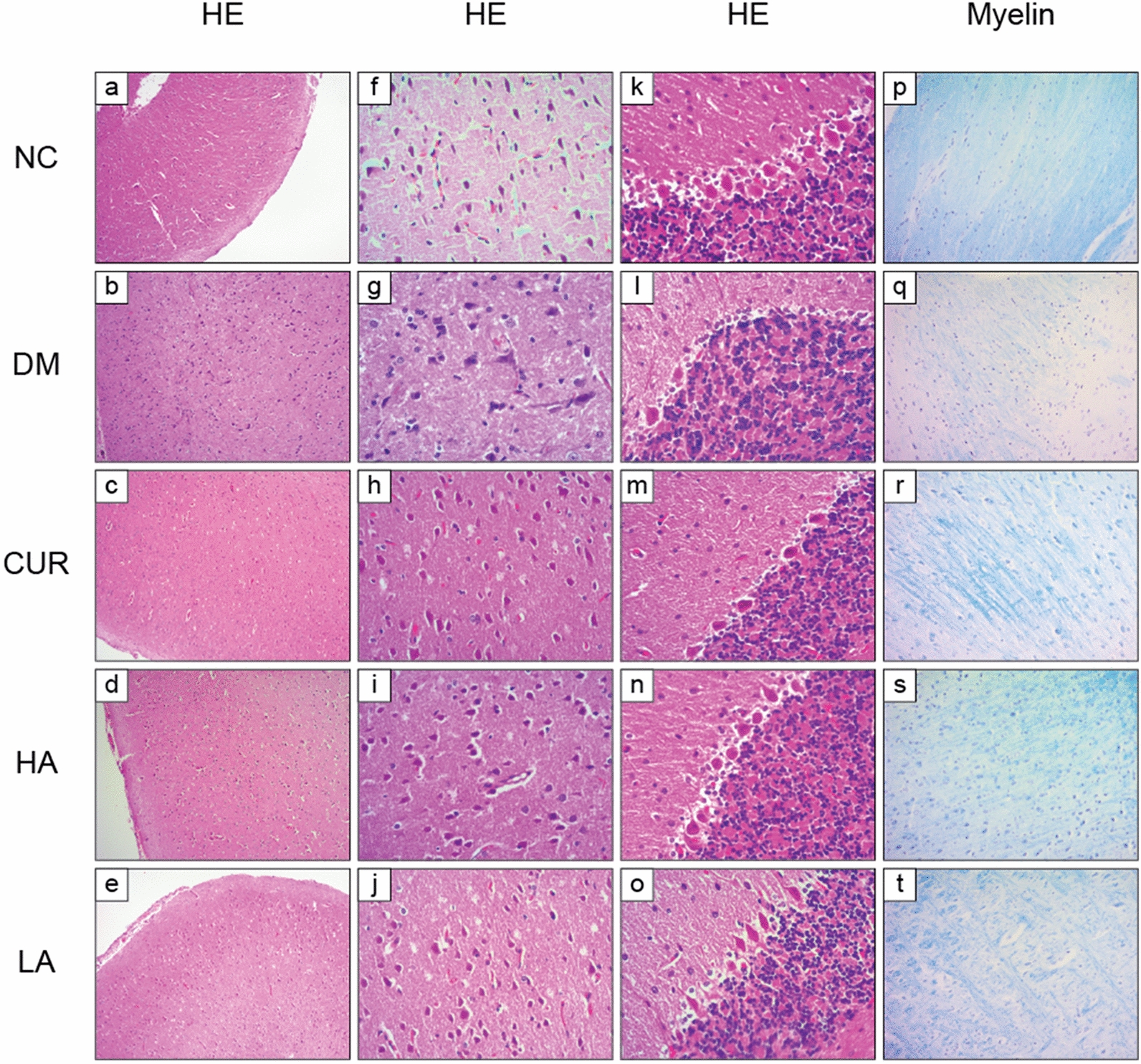
Representative microscopical images of cerebral cortex and cerebellum tissue sections from each group. Tissue sections of cerebral cortex were staind with HE staining (**a**–**e**, × 100 magnification), HE staining (**f**–**j**, × 400 magnification) and Myelin staining (**p**–**t**, × 200 magnification). Tissue sections of cerebellum were staind with HE staining (**k**–**o**, × 400 magnification)

HE staining: Upon HE staining and under a microscope, the neuronal cells in the brain of the NC group were intact and neatly arranged (Fig. 3a, f). The Purkinje cells in the cerebellum were also intact (Fig. 3k). In the DM group, we could find neuronophagia phenomenon in the brain (Fig. 3b, g). The Purkinje cells were partially lost in the cerebellar in DM group (Fig. 3l). After treatment with curcumin or A13, the above symptoms were significantly alleviated (Fig. 3c–e, h–j, m–o).

Myelin staining: Upon Myelin staining and under a microscope, in NC group, neuronal cell myelin was tight and continuous in various region of the cerebral cortex (Fig. 3p). However, myelin was reduced and lost significantly in DM group (Fig. 3q). After treatment with curcumin or A13, the changes of myelin were significantly alleviated (Fig. 3r–t).

### Role of curcumin analog A13 on NF-κB p65 pathway in the brain of diabetes rats

**Fig. 4:**
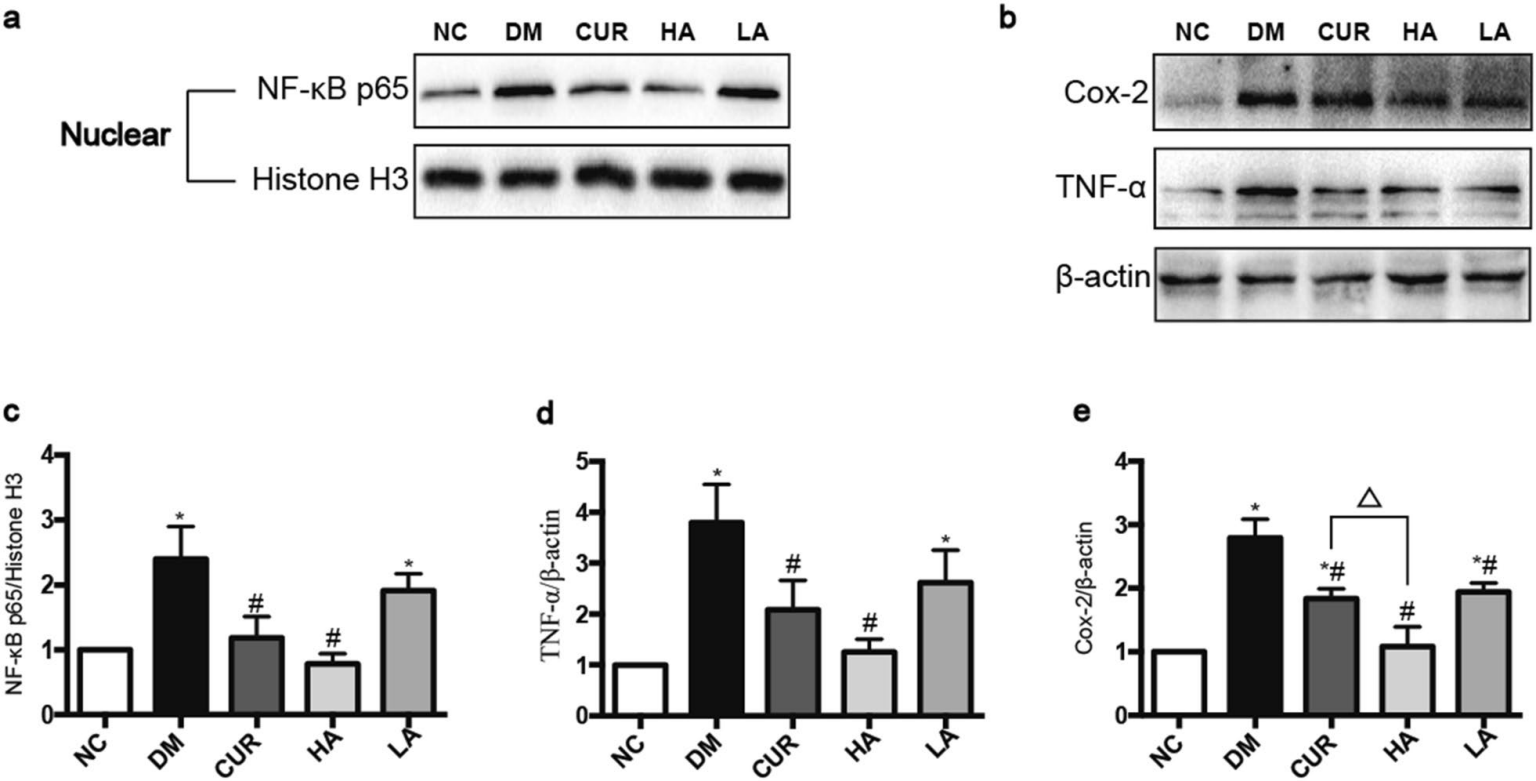
Role of curcumin analog A13 on the NF-κB p65 pathway as detected by Western blot. Expression of NF-κB p65 in nucleus (**a**, **c**); Expression of TNF-α, Cox-2 (**b**, **d**, **e**). All data are presented as mean ± SD. *P < 0.05 versus the NC group; #*P* < 0.05 versus the DM group; △*P* < 0.05 versus HA and CUR groups

The NF-κB p65 pathway was significantly activated in the brain of rats in DM group, as evidenced by increased NF-κB p65 in nuclear, TNF-α and Cox-2 in DM group (*P* < 0.05, *P* < 0.05 and *P* < 0.05 compared to the NC group). After treatment with curcumin or high-dose of A13, the levels of NF-κB p65 in nuclear (Fig. 4a, c), TNF-α significantly decreased but there was no significant change in the LA group (Fig. 4b, d). Meanwhile, curcumin and A13 could significantly reduce the level of Cox-2 (Fig. 4b, e).

### IHC staining results of NF-κB p65 and TNF-α in five groups

**Fig. 5:**
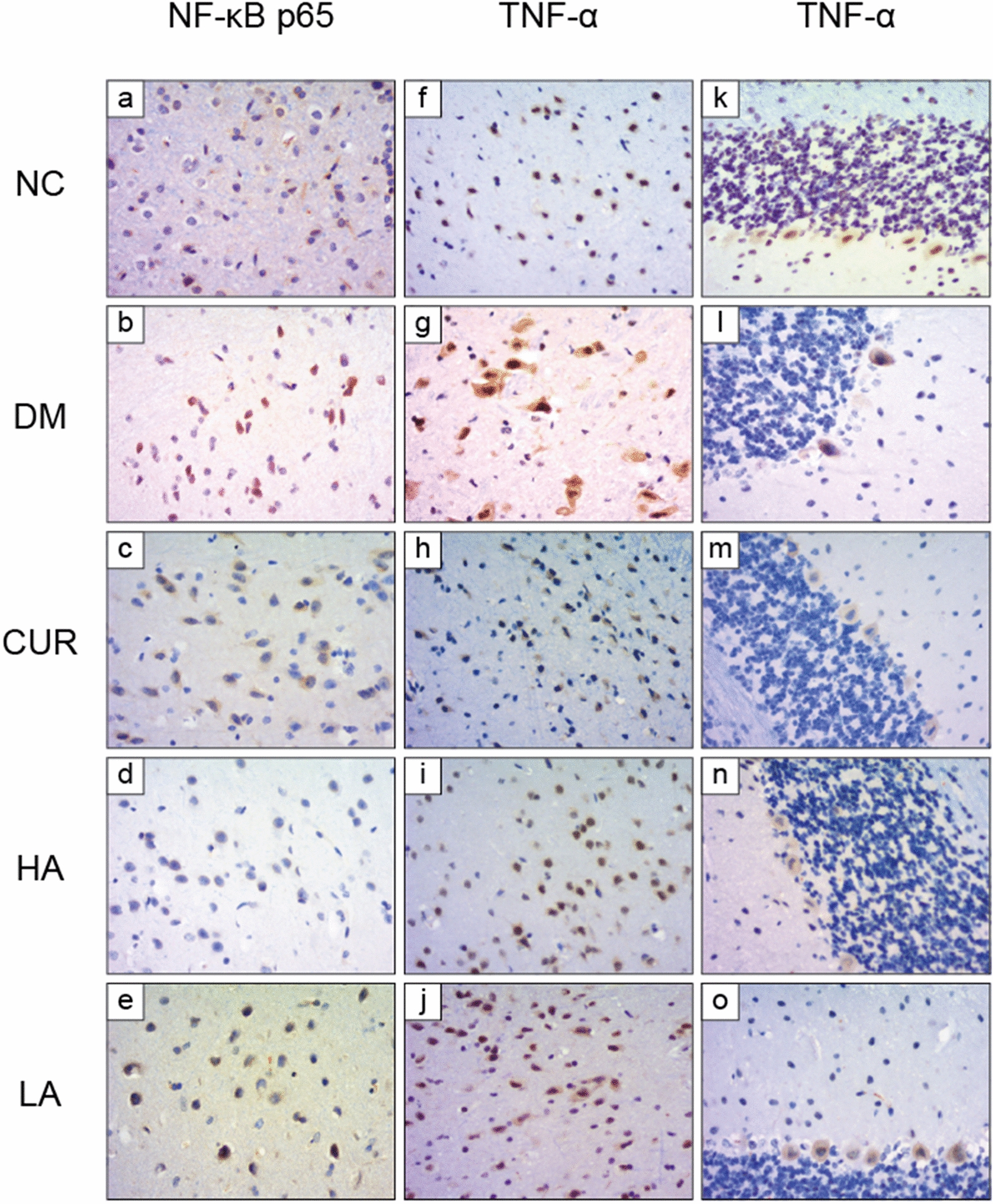
Representative microscopical images of cerebral cortex and cerebellum tissue sections from each group. Tissue sections of cerebral cortex were staind with NF-κB p65 IHC staining (**a**–**e**, NF-κB p65: × 400 magnification) and TNF-α IHC staining (**f**–**j**, TNF-α: × 400 magnification). Tissue sections of cerebellum were staind with TNF-α IHC staining (**k**–**o**, TNF-α: × 400 magnification)

Upon IHC staining and under a microscope, NF-κB p65 positive staining was localized in nucleus and cytoplasm. The total level of expression of NF-κB p65 was not significantly different in five groups. However, p65 appeared mainly in cytoplasm in the NC group (Fig. 5a) when it was positively staining in both cytoplasm and nucleus in DM group (Fig. 5b). In the other three treatment groups (Fig. 5c–e), the p65 into the nucleus was significantly reduced. Upon IHC staining and under a microscope, TNF-*α* positive staining was localized in cytoplasm. TNF-*α* protein was expressed in different levels in the brain and cerebellum in five groups. We could find that there were a small number of positive cells whose cytoplasm was stained light brown in NC group (Fig. 5f, k). However, compared with NC group, DM group had more positive cells and its cytoplasm staining is darker (Fig. 5g, l). After 8 weeks of treatment with curcumin or A13, TNF-*α*’s expression decreased significantly (Fig. 5h–j, m–o).

### Curcumin analog A13 ameliorated Oxidative stress in the brain of diabetes rats

**Fig. 6:**
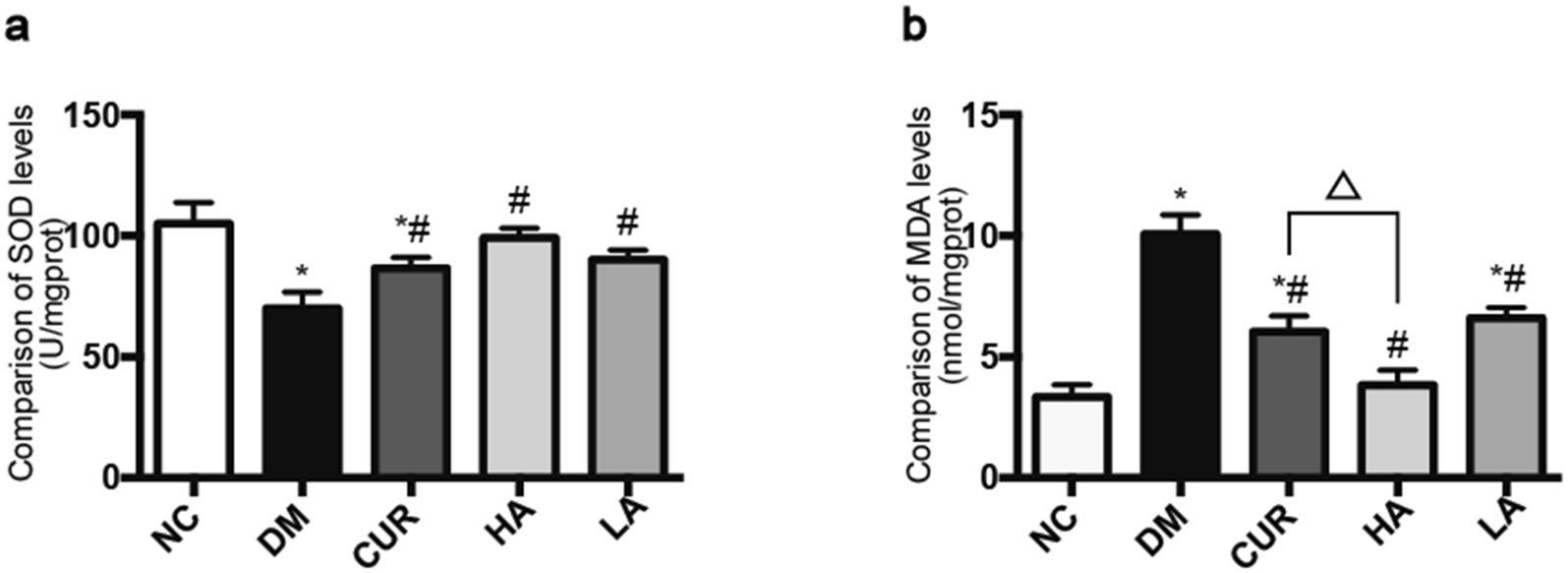
Effect of curcumin analog A13 on oxidative stress-related parameters. Activity of SOD (**a**) and MDA level (**b**). All data are presented as mean ± SD. *P < 0.05 versus the NC group; #*P* < 0.05 versus the DM group; △*P* < 0.05 versus HA and CUR groups

According to the manufacturer’s instruction, we determined the activity of SOD and the level of MDA in the tissue of brain (Fig. 6). The activity of SOD in NC group was 104.9 ± 8.8 U/mgprot. Diabetes caused the significant reduction of the activity of SOD (70.1 ± 6.6 U/mgprot). Meanwhile, the level of MDA increased from 3.4 ± 0.5 to 10.1 ± 0.8 nmol/mgprot of brain in DM group. Treatment with curcumin or A13 could restore the activity of SOD (Fig. 6a) and the level of MDA (Fig. 6b) to the level compared with NC group.

### Expression of *Nf-κb p65, Tnf-α, Cox-2 and Sod* mRNA in brain of rats

**Fig. 7:**
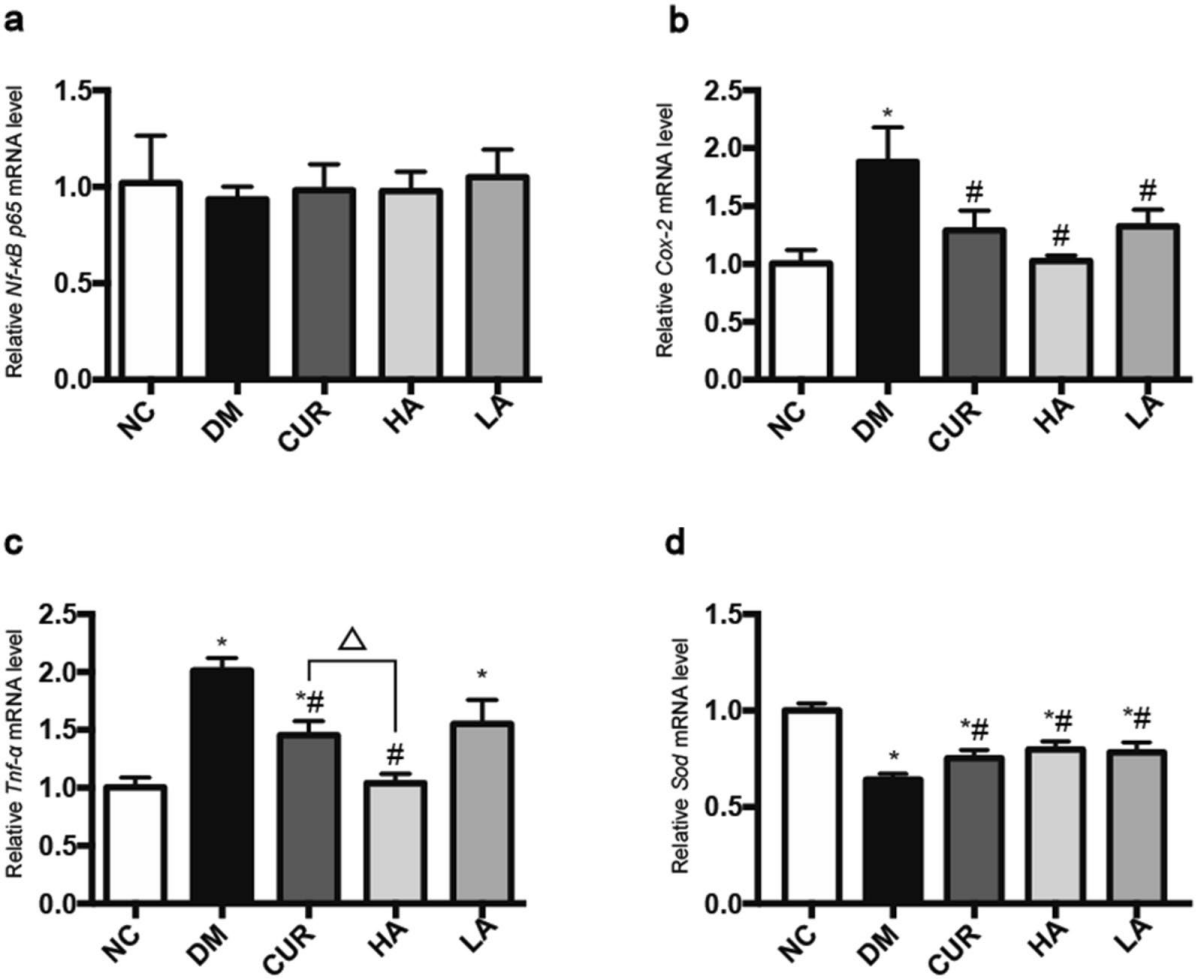
Effect of curcumin analog A13 on the NF-κB p65 pathway and oxidative stress-related parameters as detected by RT-qPCR. Level of *Nf-κb p65, Cox-2*, *Tnf-α* and *Sod* mRNA (**a**, **b**, **c**, **d**). All data are presented as mean ± SD. *P < 0.05 versus the NC group; #P < 0.05 versus the DM group; △*P* < 0.05 versus HA and CUR groups

The level of *Nf-κB p65* mRNA had no significant difference in five groups (Fig. 7a). However, compared with NC group, a significant up-regulation of *Tnf-α* mRNA and *Cox-2* mRNA expression was observed in diabetic rats (P < 0.05, P < 0.05 compared to the NC group, respectively). And curcumin or A13 could reduce the level of *Tnf-α* mRNA and *Cox-2* mRNA (Fig. 7b, c). Furthermore, the DM group presented with a lower *Sod* mRNA expression pattern when compared with NC group (*P* < 0.05). Treatment with curcumin or A13 could also alleviate this change (Fig. 7d).

## Discussion

NF-κB plays a complex role in diabetes and diabetic encephalopathy [13, 28]. In mammals, NF-κB p65 is the most common and the most studied member of NF-κB family. The activation of NF-κB p65 pathway is indicated by the phosphorylation of NF-κB p65 translocating from cytoplasm to nucleus [29]. NF-κB p65 had been shown to alter the transcription of genes which influence apoptosis, inflammation, oxidative stress and many others [30]. Strikingly, there had been a great deal of literatures to confirm the importance of NF-κB p65 and its downstream proteins including TNF-α and Cox-2 in central nervous system (CNS) [31]. The new results had confirmed that prolonged hyperglycemic internal environment could activate NF-κB p65 in the brain [32].

**Fig. 8:**
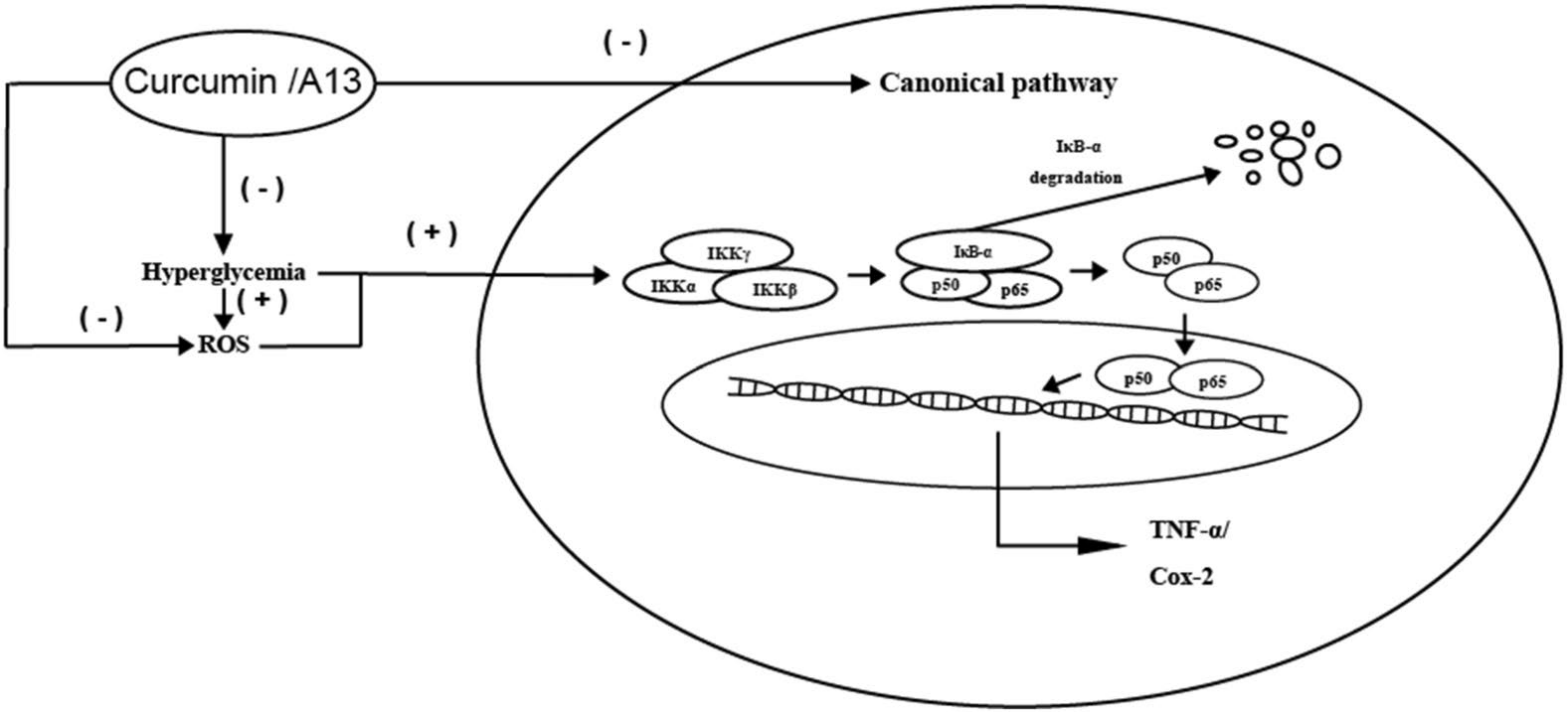
Effect of curcumin or A13 on the NF-κB p65 canonical pathway and oxidative stress-related parameters in brain of diabetic rats

The current reports from others confirmed that the expression levels of TNF-α [33], Cox-2 [34], nuclear NF-κB p65 [35] in the cerebral cortex of diabetic rats were markedly increased relative to the control rats. Meanwhile, there was some data manifesting that curcumin could decrease serum TNF-α levels in diabetes rats [36]. According to the results of Western blot and IHC staining in this study, we can conclude that p65 mainly works by entering the nucleus in the cerebral cortex of diabetic rats which is consistent with previous findings [37]. Similarly, curcumin or A13 could effectively reduce the level of p65 in the nucleus of diabetes rat. Meanwhile, this study is first to confirm that curcumin or A13 could inhibit the inflammation by decreasing the level of TNF-α and Cox-2 in the cerebral cortex in diabetic rats (Fig. 8).

Interestingly, hyperglycemic internal environment injures the brain not only by activating inflammation pathway, but also by exacerbating oxidative stress [35]. Meanwhile, reactive oxygen species (ROS) can effectively activate the NF-κB p65 pathway, suggesting that excessive oxidative stress plays a catalytic role in inflammatory response [38]. In the brain, SOD and MDA were indicated that they could regulate the glutamate tansporters-1 which injures the neuronal [39]. It has been confirmed in large number of literatures that the activity of SOD was decreased and the expression of MDA was increased in the cerebral cortex in diabetic condition [40, 41]. Curcumin has already been proven to possess antioxidant and radical scavenging properties [42]. Studies have shown that curcumin could reduce oxidative stress to take neuroprotective effects on the diabetic rat brain [43]. Consistent with previous studies, with the treatment with curcumin or A13, oxidative stress levels could be decreased (Fig. 8).

Results of RT-qPCR were consistent with those obtained by Western blot and biochemical assays analyses. The DM group had the same changes in different indexes, and curcumin or A13 also had a certain therapeutic effect.

Latest study demonstrated that oxidative stress and activation of NF-κB signaling pathway lead to demyelination in animal models [44, 45]. In this study, treatment of curcumin or A13 alleviated the damage of myelin by regulating inflammation and oxidative stress in brain of diabetic rats in this study. Remyelination in diabetic rats occurred by surviving neuroglial cells and invasive Schwann cells [46]. Diabetes inhibits the activation of neuroglial cells leading to the demyelination and the delay in remyelination processes [47]. Further study is warranted to determine the curcumin or A13 effects on remyelination in diabetic rats.

Although the efficacy of curcumin in diabetic encephalopathy is suggested by lots of studies [48, 49], we have further compared the treatment efficacy between curcumin and its analog A13 in this study. In the protein level, HA (20 mg/kg) was more effective than curcumin to decrease the level of Cox-2 and MDA (*P* < 0.05). In the gene level, HA had a better ability than curcumin to decrease *Tnf-α* mRNA (*P* < 0.05). Meanwhile, it is possible that HA (20 mg/kg) was more suitable to alleviate diabetes-induced damages in brain than LA (10 mg/kg). Further study is warranted to determine the interaction mechanisms between oxidative stress and NF-κB p65 pathway in diabetic encephalopathy.

## Conclusions

Our results revealed that the curcumin analog A13 alleviate diabetes-induced damages by regulating inflammation and oxidative stress in brain of diabetic rats. A13 may be a new potential therapeutic agent for diabetic encephalopathy. This study focused heavily on animal experiments, but there is a lack of rigorous data about the effects of A13 in cell levels. In summary, the application of A13 in diabetes complications is just at the initial stage, which needs more experiments and attempts.

## Data Availability

The datasets used and analysed during the current study are included in this published article.

## Abbreviations

DM: Diabetes mellitus
NC: Normal control
DM: Diabetes mellitus
CUR: Curcumin-treated diabetes mellitus
HA: High dose of A13-treated diabetes mellitus
LA: Low dose of A13-treated diabetes mellitus
NF-κB: Nuclear factor kappa-B
FBG: Fasting blood glucose detection
AIS: Acute ischemic stroke
ER: Endoplasmic reticulum
TNF-α: Tumor necrosis factor-α
HE: Hematoxylin–eosin
MDA: Malondialdehyde
SOD: Superoxide dismutase
IHC: Immunohistochemistry
Cox-2: Cyclooxygenase-2
CMCNa: Sodium carboxymethyl cellulose
ROS: Reactive oxygen species
CNS: Central nervous system
